# Mapping of schistosomiasis and soil-transmitted helminthiasis in the regions of Littoral, North-West, South and South-West Cameroon and recommendations for treatment

**DOI:** 10.1186/1471-2334-13-602

**Published:** 2013-12-23

**Authors:** Louis-Albert Tchuem Tchuenté, Calvine Dongmo Noumedem, Pierre Ngassam, Christian Mérimé Kenfack, Nestor Feussom Gipwe, Esther Dankoni, Ann Tarini, Yaobi Zhang

**Affiliations:** 1 National Programme for the Control of Schistosomiasis and Intestinal Helminthiasis, Ministry of Public Health, Yaoundé, Cameroon; 2 Laboratory of Parasitology and Ecology, University of Yaoundé I, Yaoundé, Cameroon; 3 Centre for Schistosomiasis and Parasitology, P.O. Box 7244, Yaoundé, Cameroon; 4 Helen Keller International, Yaoundé, Cameroon; 5 Helen Keller International, Regional Office for Africa, Dakar, Senegal

## Abstract

**Background:**

The previous nationwide mapping of schistosomiasis and soil-transmitted helminthiasis (STH) in Cameroon was conducted 25 years ago. Based on its results, mass drug administration (MDA) of praziquantel was limited to the three northern regions and few health districts in the southern part of Cameroon. In 2010, we started the process of updating the disease distribution in order to improve the control strategies. Three of the ten regions of Cameroon were mapped in 2010 and the data were published. In 2011, surveys were conducted in four additional regions, i.e. Littoral, North-West, South and South-West.

**Methods:**

Parasitological surveys were conducted in March 2011 in selected schools in all 65 health districts of the four targeted regions, using appropriate research methodologies, i.e. Kato-Katz and urine filtration.

**Results:**

The results showed significant variation of schistosomiasis and STH prevalence between schools, villages, districts and regions. *Schistosoma haematobium* was the most prevalent schistosome species, with an overall prevalence of 3.2%, followed by *S. mansoni* (3%) and *S. guineensis* (1.2%). The overall prevalence of schistosomiasis across the four regions was 7.4% (95% CI: 6.7-8.3%). The prevalence for *Ascaris lumbricoides* was 19.5% (95% CI: 18.3-20.7%), *Trichuris trichiura* 18.9% (95% CI: 17.7-20.1%) and hookworms 7.6% (95% CI: 6.8-8.4%), with an overall STH prevalence of 32.5% (95% CI: 31.1-34.0%) across the four regions. STH was more prevalent in the South region (52.8%; 95% CI: 48.0-57.3%), followed by the South-West (46.2%; 95% CI: 43.2-49.3%), the North-West (35.9%; 95% CI: 33.1-38.7%) and the Littoral (13.0%; 95% CI: 11.3-14.9%) regions.

**Conclusions:**

In comparison to previous data in 1985–87, the results showed an increase of schistosomiasis transmission in several health districts, whereas there was a significant decline of STH infections. Based on the prevalence data, the continuation of annual or bi-annual MDA for STH is recommended, as well as an extension of praziquantel in identified moderate and high risk communities for schistosomiasis.

## Background

Schistosomiasis and soil-transmitted helminthiasis (STH), two of the major neglected tropical diseases (NTDs) targeted through preventive chemotherapy, occur throughout the developing world and remain a major public health problem in the poorest communities with enormous consequences for development. Control of these diseases continues to gain momentum with increased commitment from a number of governmental and non-governmental donors to provide the funding and from pharmaceutical companies to donate the anthelminthic drugs. In Cameroon, more than 5 million people are at risk of infection with schistosomiasis, and 2 million persons are currently infected. STHs are widely distributed all over the country, and it is estimated that more than 10 million people are infected with intestinal worms [1]. The national epidemiological survey conducted in 1985–1987 showed the occurrence of three species of schistosomes: *Schistosoma haematobium*, *S. mansoni* and *S. guineensis* (formerly *S. intercalatum* Lower Guinea strain [2,3]); and three major species of STH: *Ascaris lumbricoides*, *Trichuris trichiura* and *Necator americanus*. The highest transmission levels of schistosomiasis occurred in the savannah areas of the northern Cameroon, whereas STHs were more prevalent in the southern forest part of the country [4-6]. School age children were the most infected, and polyparasitism was very frequent; with a largest proportion of children carrying at least 2 species of parasites [7].

Cameroon adopted a strategic plan for the control of schistosomiasis and STH in 2004. Starting with a very limited budget, the control programme gradually mobilized national and international partners to enable a rapid scaling-up of activities to encompass all ten regions in 2007. Since then, national deworming campaigns have been implemented annually. School age children were treated with mebendazole nationwide, whereas praziquantel was distributed only in high endemic areas for schistosomiasis [1]. Since 2009, the control of schistosomiasis and STH in Cameroon has been integrated with other national control programs, i.e. onchocerciasis, lymphatic filariasis and trachoma, to form an integrated national NTD control program. The national NTD program has developed a national plan for co-implementation of different control interventions and co-administration of several drugs, including praziquantel, ivermectin, mebendazole and albendazole in a coordinated effort. This integrated approach is used to maximize cost-effectiveness and efficiency. The integrated national NTD control program receives support from the United States Agency for International Development (USAID) through its NTD Control Program, currently ENVISION program, managed by RTI International [8].

Knowledge of the distribution of the targeted NTDs is essential for developing an adequate implementation strategy and designing drug packages for co-administrations. One of the major efforts of the integrated national NTD control program in Cameroon was to update the disease distribution information by conducting the national mapping of diseases, as the baseline data for schistosomiasis and STH in Cameroon were outdated as they were collected 25 years ago [4,5]. It is well known that the transmission of these diseases is dynamic over time, particularly after years of treatment and other health interventions [9]. The first phase of the mapping was conducted in Centre, East and West regions, and the results have been published [10]. The second study phase targeted four of the ten regions of Cameroon, i.e. Littoral, North-West, South and South-West. The present paper reports the outcome of the mapping exercises, compares the current situation with the baseline data from 1980s, and provides recommendations for the control of schistosomiasis and STH in these regions.

## Methods

### Ethical statement

The study was approved by the National Ethics Committee of Cameroon (Nr 082/CNE/DNM/09), and was a public health exercise through the Ministry of Health and the Ministry of Education. Parasitological surveys were conducted in schools with the approval of the administrative authorities, school inspectors, directors and teachers. Information about the national programme for the control of schistosomiasis and STH, and the objectives of the study were explained to the schoolchildren and to their parents or guardians from whom informed consent was obtained. Children willing to participate were registered. Each child was assigned an identification number and data collected were entered in a database. No identification of any child can be revealed upon publication. Children were treated during the MDA campaign implemented by the national control programme.

### Study area

Cameroon is divided into a three-tiered administrative system including 10 regions at the first level, 58 divisions (*departments*) at the second level, and 360 sub-divisions (*arrondissements*) at the third level. The population of Cameroon was estimated to be 19,406,100 inhabitants in 2010. Population density shows marked variation across the country, ranging from a mean of 7.4 inhabitants/km^2^ in the East region to 141.5 inhabitants/km^2^ in the Littoral region. School age children account for 28% of the country population and are estimated at 5,433,708 [11]. The health system in Cameroon is decentralized and organized into central, regional and district levels. There are 179 health districts. The four regions targeted for mapping, i.e. Littoral, North-West, South and South-West, have a total of 65 health districts and are located in the southern forest area of the country. These regions are subdivided in 19, 18, 18 and 10 health districts, respectively.

### Sampling and data collection

A stratified random-cluster sampling procedure, with the 5^th^ grade of school children as the basic sampling unit, was used in the previous mapping of schistosomiasis and STH in Cameroon, conducted in 1985–1987 [4,12]. In that survey, sampling units were selected from a complete list of the public and private primary schools according to regions (formerly provinces) and divisions. Schools were selected at random with chances of being selected proportional to the school population without consideration of an even spatial coverage [4,12]. In order to assess the current levels of infections, in the current survey the schools were selected using the list of villages and schools, taking into account the ecological zones, the risk factors and the prior local knowledge for schistosomiasis transmission according to the new survey guidelines [13,14]. Schools were selected in all health districts of the four-targeted regions of Cameroon, with a relatively even spatial coverage. Due to the financial limitations, one or two primary schools (depending on the district's size, population density, and ecological zones) were selected per health district. In each district, schools with previously known higher prevalence or located in areas of high risk of transmission were selected in priority. The geographical co-ordinates of each of the sampled schools were recorded with global positioning system (GPS) devices. The study was conducted in March 2011.

In the 1985–1987 study, a 10 ml urine sample was examined for *S. haematobium*, and a single Kato-Katz slide was examined for *S. mansoni, S. guineensis* and STH infections [4,12]. In the current study, in each school, urine and stool samples were collected from 50 children, approximately half boys and half girls. Children were preferentially selected from the 5^th^ and 6^th^ grades (8–19 years old, mean 12 years), and then from other grades where the number of children in the 5^th^ and 6^th^ grades was fewer than 50. This resulted in an age range from 5–19 years in the selected children with 81% being 9–13 years old. The samples were collected in 60 ml plastic screw-cap vials, between 10.00 and 14.00 hours. The samples were transported to the laboratory for examination the same day. Only few samples were preserved with sodium azide [4,12], and examined within the following two days. In the laboratory, each urine sample was agitated to ensure adequate dispersal of eggs, 10 ml of urine were filtered through a Nucleopore® filter, and the filters were examined by microscopy for the presence of schistosome eggs. Stool samples were examined by a single thick smear technique using a 41.7 mg Kato-Katz template. Each slide was read twice; immediately after slide preparation for hookworm eggs, and the following day for schistosome and other STH eggs. Parasitic infections were recorded; number of eggs for each parasite was counted; and intensity of infection was calculated and expressed as eggs per gram of feces (epg) or eggs per 10 ml of urine (egg/10 ml).

### Data analysis

The different parasitological data were entered into Excel and cleaned for entry errors at the epidemiological unit of the Centre for Schistosomiasis & Parasitology in Yaounde, Cameroon. The data were subsequently imported into SPSS (IBM, Version 19) for statistical analysis. The Complex Samples Crosstabs procedure was used for calculating the prevalence and the Descriptive procedure for calculating the intensity of infections taking into account the cluster nature of schools with districts as strata and schools as clusters and including the finite population correction assuming equal probability sampling without replacement. Sample weighting was applied for each district according to the population size of the school age children (5–15 years old) in each district, i.e. the weight for each district was calculated as the ratio of the expected number of children to be surveyed (= number of school age children in a district × overall proportion of the children actually sampled) and the number of children actually surveyed in each district. The 95% confidence intervals (CIs) for prevalence were calculated using the Wilson score method without the continuity correction after adjusting for sample weighting [15], using the CI calculator (available: http://vl.academicdirect.org/applied_statistics/binomial_distribution/ref/CIcalculator.xls). Arithmetic mean abundance of infection with 95% CIs for different parasite species were calculated including all children examined including both the positives and negatives [16-18]. When calculating the overall prevalence of schistosomiasis, only those children with valid data entries on both urine and stool examinations were included. The Chi-square test using the Complex Samples Crosstabs procedure was used to investigate the relationship between prevalence of infections and sex, age groups, districts and regions. The Kruskal-Wallis test was used to compare the differences in abundance of infection. The levels of endemicity of schistosomiasis and STH and the degrees of intensity of individual infections were categorized according to the World Health Organization (WHO) recommendations [19,20]. A geographical information system (GIS) software ArcGIS (ESRI Inc., Version 9.2) was used to plot the point prevalence of the infections for each surveyed school on a map.

## Results

A total of 81 schools were surveyed: 28 schools in the Littoral region, 18 schools in the North-West region, 17 schools in the South-West region and 18 schools in the South region. A total of 4,130 pupils (2,030 males and 2,100 females) from these 81 schools were registered and included in the study. Of these children registered, 4,091 (99.03%) and 4,034 (97.65%) provided urine and stool samples, respectively. There were 3,999 children who provided both urine and stool samples. The mean age (± standard deviation) of children examined was 10.94 ± 2.07 years.

### Schistosomiasis

#### Prevalence

Table 1 summarizes the survey results for individual parasites in each region. The results are shown as prevalence and intensity of infections together with 95% CI.

**Table 1:** Adjusted prevalence and abundance of parasitic infections (95% CI) in school children in Littoral, North-West, South and South-West regions in Cameroon

	**Overall no of persons examined**	***S. haematobium***	***S. guineensis***	***S. mansoni***	***A. lumbricoides***	**Hookworm**	***T. trichiura***
**Prevalence (%)**							
*Overall*	4130	3.2 (2.7 - 3.8)	1.2 (0.9 - 1.6)	3.0 (2.5 - 3.6)	19.5 (18.3 - 20.7)	7.6 (6.8 - 8.4)	18.9 (17.7 - 20.1)
*By region*							
Littoral	1559	1.8 (1.2 - 2.6)	0.3 (0.1 - 0.8)	2.0 (1.4 - 2.8)	5.8 (4.7 - 7.2)	0.3 (0.1 - 0.8)	10.4 (8.8 - 12.1)
North West	898	0.6 (0.3 - 1.3)	0.0 (0–0.34)	4.1 (3.1 - 5.4)	28.1 (25.6 - 30.8)	9.8 (8.2 - 11.6)	6.3 (5.1 - 7.9)
South	783	0.0 (0.0 - 0.85)	0.2 (0.0 - 1.3)	0.3 (0.0 - 1.3)	28.5 (24.5 - 32.9)	7.1 (5.0 - 9.9)	45.4 (40.7 - 50.0)
South West	890	9.3 (7.7 - 11.2)	4.2 (3.1 - 5.6)	4.3 (3.3 - 5.8)	24.3 (21.7 - 27.0)	15.0 (13.0 - 17.3)	32.9 (30.0 - 35.8)
*By sex*							
Male	2026	3.3 (2.6 - 4.1)	1.2 (0.8 - 1.8)	2.9 (2.2 - 3.7)	19.6 (17.9 - 21.4)	8.1 (7.0 - 9.5)***	19.9 (18.2 - 21.7)***
Female	2100	3.1 (2.4 - 3.9)	1.3 (0.9 - 1.9)	3.1 (2.5 - 4.0)	19.4 (17.7 - 21.2)	7.0 (6.0 - 8.2)	17.9 (16.3 - 19.6)
							
Abundance of infection (epg)*							
*Overall*		1.8 (1.7 - 2.0)	3.3 (0.3 - 6.2)	9.3 (7.0 - 11.6)	1880.4 (1629.4 - 2131.4)	25.9 (21.1 - 30.7)	240.5 (152.0 - 329.0)
0 epg (%)	-	96.8 (96.2 - 97.3)	98.1 (97.7 - 98.5)	95.6 (94.9 - 96.2)	79.8 (78.5 - 81.0)	91.5 (90.6 - 92.4)	76.2 (74.9 - 77.5)
Light (%)	-	2.5 (2.0 - 3.0)	0.9 (0.6 - 1.2)	2.2 (1.8 - 2.7)	12.1 (11.1 - 13.2)	8.1 (7.3 - 9.0)	19.3 (18.1 - 20.5)
Moderate (%)	-	-	0.7 (0.5 - 1.0)	1.2 (0.9 - 1.6)	6.8 (6.1 - 7.6)	0.2 (0.1 - 0.5)	3.2 (2.8 - 3.8)
Heavy (%)	-	0.7 (0.5 - 1.1)	0.3 (0.2 - 0.5)	1.0 (0.7 - 1.4)	1.3 (1.0 - 1.7)	0.1 (0.0 - 0.2)	1.3 (1.0 - 1.7)
*By region*							
Littoral	1559	0.38 (0.0 - 0.80)	0.54 (0.0 - 1.14)	3.96 (0.0 - 8.63)	935.73 (328.13 - 1543.34)	0.55 (0.0 - 1.20)	73.41 (8.42 - 138.40)
North West	898	0.01 (0.01 - 0.02)	-	18.89 (18.60 - 19.18)	2677.54 (2648.30 - 2706.78)	39.34 (38.20 - 40.48)	9.34 (9.17 - 9.52)
South	783	0.08 (0.0 - 0.26)	0.27 (0.03 - 0.52)	7.26 (0.0 - 22.46)	4469.64 (3843.42 - 5095.85)	20.30 (0.0 - 40.63)	1586.33 (845.51 - 2327.15)
South West	890	6.62 (6.45 - 6.79)	11.88 (0.52 - 23.24)	6.65 (6.44 - 6.85)	1153.43 (730.84 - 1576.02)	47.32 (31.06 - 63.57)	145.87 (53.03 - 238.71)
*By sex*							
Male	2026	2.44 (2.26 - 2.61)	3.93 (0.0 - 10.38)	9.28 (5.78 - 12.78)	2001.61 (1754.42 - 2248.79)	28.00 (22.17 - 33.84)**	239.10 (161.65 - 316.55)
Female	2100	1.28 (1.11 - 1.45)	2.66 (2.09 - 3.23)	9.33 (7.02 - 11.63)	1766.80 (1488.29 - 2045.31)	23.91 (19.49 - 28.32)	242.15 (123.40 - 360.89)

Note: *eggs/10 ml urine for *S. haematobium* intensity of infection; light, moderate and heavy infections was categorized according to the WHO recommendations [20]; *S. guineensis* infection was categorized using the recommendations for *S. mansoni*. **p < 0.05; ***p < 0.01.

**Figure 1:**
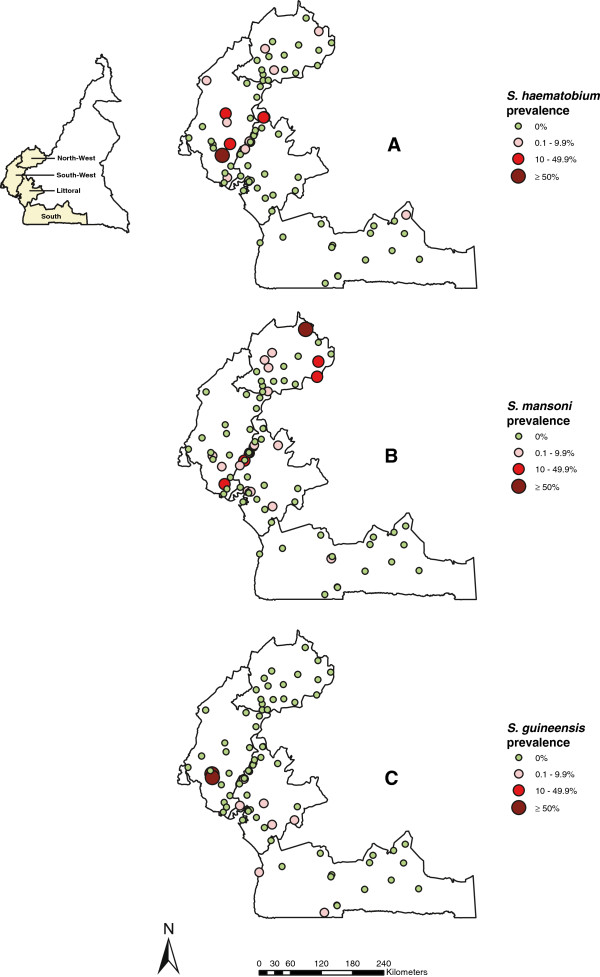
**Prevalence of schistosomiasis by school in the four surveyed regions of Cameroon. (A)***S. haematobium*, **(B)***S. mansoni*, and **(C)***S. guineensis*.

*S. haematobium* infected children were found in 19 of the 81 schools investigated, with an average prevalence of 3.2% ranging from 0% to 82% across the four regions. *S. mansoni* was found in 22 schools with an average prevalence of 3%, ranging from 0% to 86% across the four regions. *S. guineensis* was found in 9 schools, with relatively low prevalence of 1.2%, varying from 0% to 81.6%. The highest prevalence of schistosomiasis, 86% for *S. mansoni*, was found in Fako health district, North-West region. The point prevalence distribution of schistosomiasis in all surveyed schools is shown in Figure 1. There was an evident difference of schistosome infections between surveyed schools. The majority of schools were negative for schistosomiasis.

Among the four regions surveyed, *S. haematobium* infection was highest in the South-West region with an average prevalence of 9.3% (95% CI: 7.7-11.2%), and was relatively low in other three regions (0.0-1.8%) (Table 1). *S. mansoni* infection prevalence was relatively low in all four regions (0.3-4.3%). Apart from the South-West region, low levels of *S. guineensis* infection were found in the Littoral, South and North-West regions. The overall prevalence of schistosomiasis (including all three species) across the four regions was 7.4% (95% CI: 6.7-8.3%): 18.0% (95% CI: 15.8-20.6%) in the South-West region, 4.7% (95% CI: 3.7-6.1%) in the North-West region, 4.2% (95% CI: 3.2-5.3%) in the Littoral region and 0.5% (95% CI: 0.1-1.7%) in the South region (Table 2). There was no significant difference of schistosomiasis prevalence between boys and girls (Chi-square test, χ^2^ = 0.13, df = 1, p > 0.05) in the four regions.

**Table 2:** Comparison of prevalence (95% CI) of schistosomiasis and STH between 1980s and 2011

	**No of schools surveyed in 1985-87**	**No of children examined in 1985-87**	**Results from 1985-1987**	**No of schools surveyed in 2011**	**No of children examined in 2011**	**Results in 2011**	**Percentage difference (%)**
**Schistosomiasis prevalence (%)**							
*Overall*	75	4760	4.3 (3.8 - 4.9)	81	4130	7.4 (6.7 - 8.3)	72.1
*By region*							
Littoral	38	1706	8.6 (7.3 - 10.0)	28	1559	4.2 (3.2 - 5.3)	-51.2
North West	7	1149	1.5 (0.9 - 2.4)	18	898	4.7 (3.7 - 6.1)	213.3
South	29	858	2.2 (1.4 - 3.4)	18	783	0.5 (0.1 - 1.7)	-77.3
South West	1	1047	2.2 (1.5 - 3.3)	17	890	18.0 (15.8 - 20.6)	718.2
**STH prevalence (%)**							
*Overall*	75	4760	88.9 (88.0 - 89.7)	81	4130	32.5 (31.1 - 34.0)	-63.4
*By region*							
Littoral	38	1706	92.7 (91.6 - 93.7)	28	1559	13.0 (11.3 - 14.9)	-86.0
North West	7	1149	72.3 (69.7 - 74.8)	18	898	35.9 (33.1 - 38.7)	-50.3
South	29	858	97.9 (96.8 -98.7)	18	783	52.8 (48.0 - 57.3)	-46.1
South West	1	1047	91.1 (89.3 - 92.7)	17	890	46.2 (43.2 - 49.3)	-49.3

#### Abundance of infection

The arithmetic mean abundance of infection in the four regions for each species of schistosomes is shown in Table 1. The egg counts ranged from 0 to 4,392 epg for intestinal schistosomiasis, and from 0 to 3,609 eggs/10 ml for urinary schistosomiasis. The arithmetic mean abundance of infection was 9.3 epg (95% CI: 7.0-11.6 epg) for *S. mansoni*, 1.8 eggs/10 ml (95% CI: 1.7-2.0 eggs/10 ml) for *S. haematobium*, and 3.3 epg (95% CI: 0.3-6.2 epg) for *S. guineensis*. The North-West region was most heavily infected with *S. mansoni* (18.9 epg) and the South-West region with *S. haematobium* (6.6 eggs/10 ml) and *S. guineensis* (11.9 epg). It appears that infections were light (<100 epg or <50 eggs/10 ml) in the majority of schools, with only 2.2% moderate or heavy *S. mansoni* infections and 0.7% heavy *S. haematobium* infections across the four regions (Table 1). The difference in abundance of infections with any individual schistosome species between boys and girls was not significant (Kruskal Wallis test, H_Sh_ = 0.727, H_Sm_ = 0.15, H_Sg_ = 0.073, df = 2, p > 0.05).

### Soil-transmitted helminthiasis

#### Prevalence

**Figure 2:**
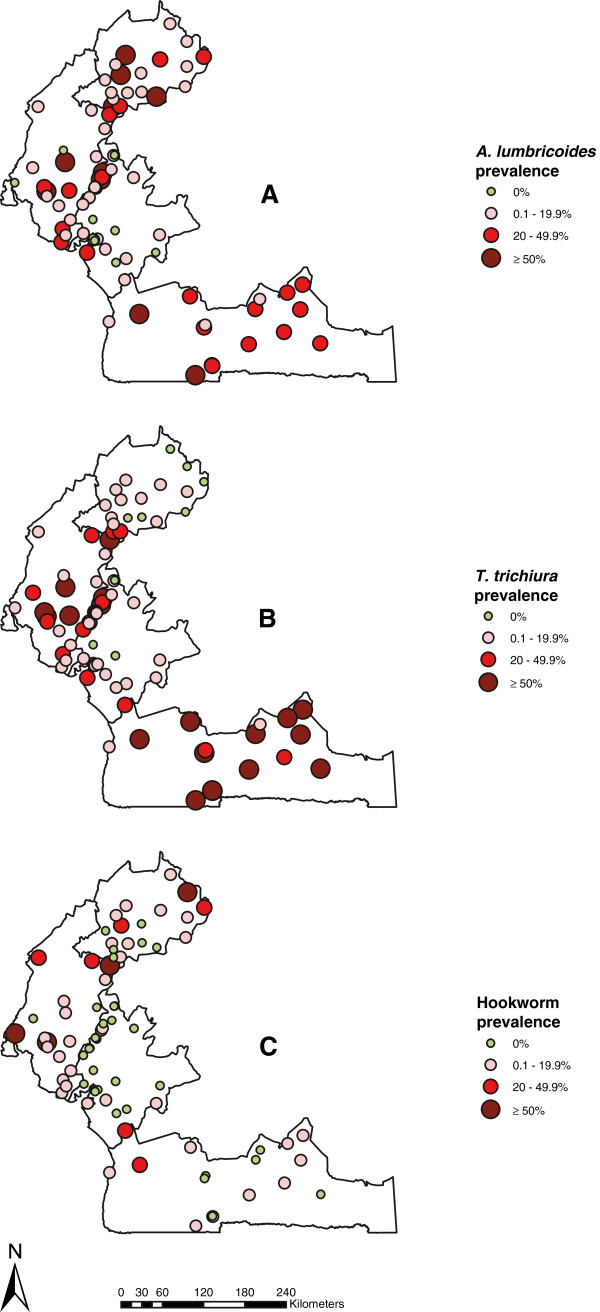
**Prevalence of soil-transmitted helminthiasis by school in the four surveyed regions of Cameroon. (A)***A. lumbricoides*, **(B)***T. trichiura*, and **(C)** Hookworm infections.

As shown in Table 1, *A. lumbricoides* was the most prevalent STH with an overall prevalence of 19.5% (95% CI: 18.3-20.7%), followed by *T. trichiura* with an overall prevalence of 18.9% (95% CI: 17.7-20.1%) and hookworm with 7.6% (95% CI: 6.8-8.4%) across the four regions. There was a significant difference between regions among individual STH species (Chi-square test, χ_Al_^2^ = 259.4, df_Al_ = 2.4; χ_Tt_^2^ = 523.5, df_Tt_ = 2.2; χ_Hw_^2^ = 196.5, df_Hw_ = 1.8; p < 0.001) and a significant geographical heterogeneity in STH distribution among schools surveyed across the four regions (Figure 2). There was not much difference in *A. lumbricoides* prevalence between boys and girls (Chi-square test, χ^2^ = 0.022, df = 1, p > 0.05), though significantly different for *T. trichiura* (Chi-square test, χ^2^ = 2.634, df = 1, p < 0.01) and hookworms (Chi-square test, χ^2^ = 1.909, df = 1, p < 0.01).

The overall prevalence of STH (including all three species) across the four regions was 32.5% (95% CI: 31.1-34.0%): 13.0% (95% CI: 11.3-14.9%) in the Littoral region, 35.9% (95% CI: 33.1-38.7%) in the North-West region, 52.8% (95% CI: 48.0-57.3%) in the South region and 46.2% (95% CI: 43.2-49.3%) in the South-West region (Table 2). There was significant difference between regions (Chi-square test, χ^2^ = 419.3, df = 2.3, p < 0.001). There was no significant difference in overall prevalence of STH between boys (32.9%; 95% CI: 30.8 - 35.0%) and girls (32.2%; 95% CI: 30.2 - 34.3%) (Chi square test, χ^2^ = 0.21, df = 1, p > 0.05).

#### Abundance of infection

*A. lumbricoides* infection was the most abundant infection across the four regions, with an arithmetic mean abundance of 1880.4 epg (95% CI: 1629.4-2131.4 epg), followed by *T. trichiura* infection with an arithmetic mean abundance of 240.5 epg (95% CI: 152.0-329.0 epg), while hookworm infection was light or moderate (Table 1). The maximum egg count for *A. lumbricoides* was 166,536 epg, *T. trichiura* 142,152 epg, and hookworm 15,432 epg. Corresponding to the prevalence data, the South region was most heavily infected with *A. lumbricoides* and *T. trichiura* (Table 1). Boys were slightly more heavily infected with hookworms than girls (Kruskal Wallis test, H = 5.179, df = 1, p < 0.05) (Table 1).

#### Comparison of 1985–1987 and 2011 data

**Figure 3:**
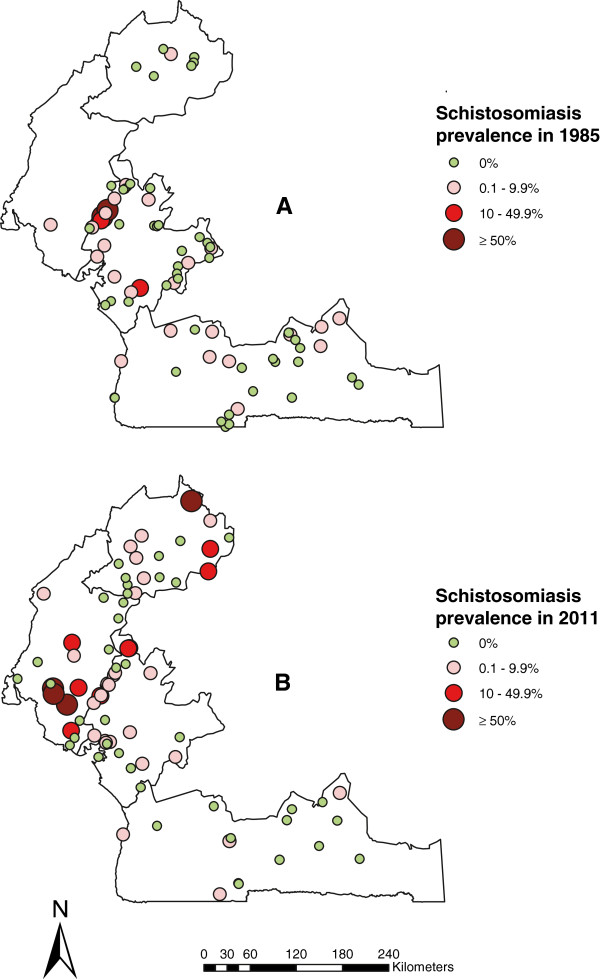
**Comparative maps of the overall schistosomiasis prevalence in the four surveyed regions of Cameroon.** Prevalence distribution in 1985–1987 **(A)** and in 2011 **(B)**.

The current schistosomiasis and STH distribution in 2011 was compared with those obtained between 1985–1987, using the overall schistosomiasis and STH prevalence (Table 2). The baseline level of infections for schistosomiasis and STH was published previously [4-6]. The prevalence distribution of schistosomiasis in 1985–1987 and in 2011 is shown in Figure 3, with the prevalence categorized according to the WHO prevalence thresholds [20]. The data show that the overall endemic areas of schistosomiasis did not change significantly; however, there was an increase in the number of high transmission foci of schistosomiasis in several health districts, particularly, in the South-West region, where the overall prevalence increased from 2.2% to 18.0%.

**Figure 4:**
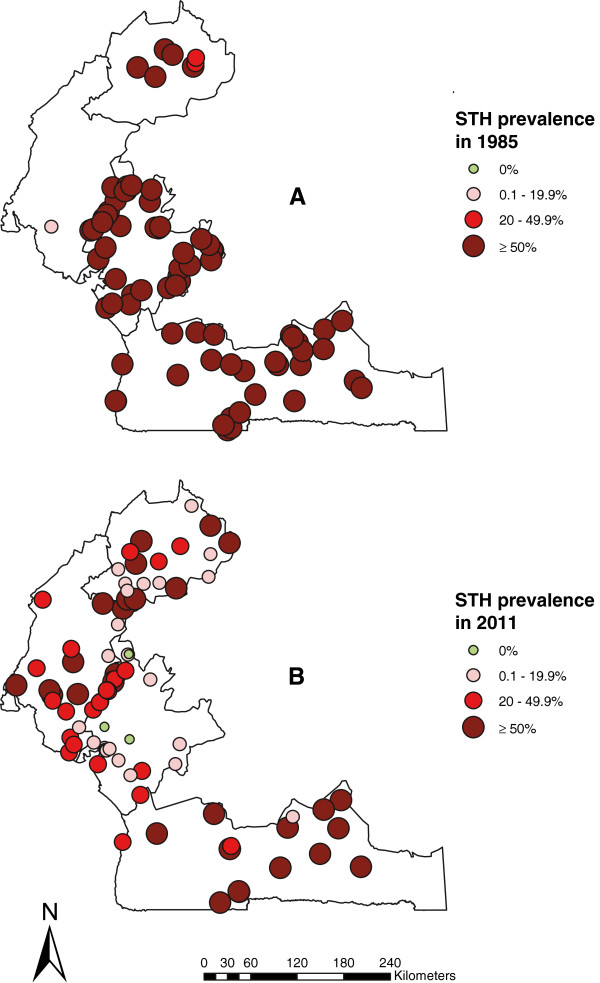
**Comparative maps of the overall soil-transmitted helminthiasis prevalence in the four surveyed regions of Cameroon.** Prevalence distribution in 1985–1987 **(A)** and in 2011 **(B)**.

The prevalence distribution of STH in 1985–1987 and in 2011 is shown in Figure 4. There was a clear and noticeable reduction of STH prevalence in all four regions. Indeed, the overall STH prevalence declined from 92.7%, 72.3%, 97.9% and 91.1% in 1985–1987 to 13%, 35.9%, 52.8% and 46.2% in 2011 in the regions of Littoral, North-West, South and South-West, respectively (Table 2). The reduction of STH was greatest (by 86%) in the Littoral region in comparison to the three other regions.

## Discussion

The mapping results in the Littoral, North-West, South and South-West regions of Cameroon showed that schistosomiasis was endemic in all four regions, but with relatively low level of endemicity. Among the 81 schools surveyed, infection was found in only 19, 22 and 9 schools for *S. haematobium*, *S. mansoni* and *S. guineensis*, respectively. The average prevalence in each region was below 10% for all three schistosome species. However, due to the typical nature of focal transmission of schistosomiasis, moderate or high endemicity was found in 12 schools across the 81 schools surveyed in the four regions, with overall schistosomiasis prevalence (including all three species) up to 86%. When comparing our results with the previous nationwide data collected in 1985–1987 by Ratard et al. [4], it appears a slight increase of the number of high transmission foci of schistosomiasis. This is not surprising given the fact that no mass drug administration (MDA) with praziquantel had been implemented in these health districts since the last mapping survey. It is also noted that the main purpose of the current survey was to map out the distribution of schistosomiasis in these regions so that the implementation of the MDA intervention could be planned. Therefore, in contrast to the 1985–87 survey, the current survey took into consideration a relatively even spatial coverage to include all health districts. Also, ecological and risk factors and prior knowledge were considered during selection.

The overall level of schistosomiasis endemicity in these regions was in line with our expectation, and was similar to the endemicity level found in other southern regions [10]. Indeed, based on the 1985–1987 mapping, the highest transmission level of schistosomiasis is in the northern part of Cameroon [4,21], and MDA with praziquantel had been primarily focused in the three northern regions, whereas in the southern part of the country regular treatment with praziquantel was implemented only in those very few health districts highly endemic for schistosomiasis [10]. Apart from two districts (i.e. Loum in the Littoral region and Mbongue in the South-West region), none of the other districts in the four regions investigated in the present study had received regular treatment with praziquantel before this survey. Further to the key outcomes and recommendations in the last publication from the mapping in the Centre, East and West regions [10], in future deworming campaigns, the distribution of praziquantel should be undertaken in all endemic health districts to include all school age children according to the WHO roadmap for schistosomiasis elimination and the preventive chemotherapy guidelines for schistosomiasis in school age children [20,22,23]. Considering the overall low endemicity of schistosomiasis in the majority of these health districts, treatment will be conducted at district level in rural zones, whereas in urban settings treatment will be focused in those sub-districts with high prevalence spots of schistosomiasis.

For STH, the current mapping showed an overall significant reduction of infection prevalence in all four regions investigated in comparison to previous mapping data collected in 1985–1987 [5,6,21], similar to the recent mapping in the Centre, East and West regions of Cameroon [10]. The overall STH prevalence declined by 46-86% from 1985–87 to 2010 in the four regions surveyed. The decline was greatest in the Littoral region compared to the three other regions. As previously discussed [10], school age children in Cameroon have been dewormed with mebendazole nationwide in all 179 health districts since 2007. These results clearly illustrated the positive impact of the school-based deworming campaigns implemented annually by the Ministry of Public Health, through the National Programme for the Control of Schistosomiasis and Intestinal Helminthiasis. In addition to the positive impact of repeated treatment with mebendazole, the reduction in STH prevalence may have also benefited from the ivermectin MDA implemented in onchocerchiasis endemic communities. Despite the observed significant reduction of STH infections, the overall STH prevalence was still over the treatment intervention threshold (>20%) in North-West, South and South-West regions and in many communities in the Littoral region, and intensities of *A. lumbricoides* and *T. trichiura* infections were still relatively high. Therefore, the national control program should continue implementing annual deworming of school age children in all endemic districts of these regions. In addition, preschool children, women of childbearing age and adults at high-risk in certain occupations should also be treated, according to WHO recommendations [20].

Over the past few decades, significant progress has been made in the control of schistosomiasis, STH and other NTDs. WHO recommends comprehensive control measures for the control of NTDs including preventive chemotherapy, intensified and innovative disease management, vector and intermediate host control, veterinary public health at the human-animal interface, and provision of safe water, sanitation and hygiene [9,22]. However, the current funding almost exclusively focuses on preventive chemotherapy with specific anthelminthic drugs that can safely be co-administered in co-endemicity situations, e.g. praziquantel and albendazole/mebendazole for the control of schistosomiasis and STH. In order to appropriately determine where treatment for which disease is required, accurate mapping of the different NTDs is a pre-requisite. Based on infection prevalence, communities can then be classified into low, moderate and high-risk categories according to WHO specific disease thresholds and the appropriate treatment regimen applied [20]. The current mapping results provided a foundation for such program planning.

Finally, the results of the present study highlight the need of implementing praziquantel MDA in the endemic health districts in the four regions for the control and elimination of schistosomiasis. Based on the results, medicines were procured and, within the integrated national NTD control program, praziquantel MDA was extended in 11 of the 65 health districts in the four targeted regions; i.e. 3 health districts in the Littoral region, 3 in the North-West and 5 in the South-West. The results also highlight the need of continued deworming for STH infections in all 65 health districts of these regions. This effort will be coordinated with the lymphatic filariasis MDA using albendazole when it starts. The results also contribute to updating global information resource on the distribution of schistosomiasis and STH, recently developed as an open-access database [24].

## Conclusions

This study showed a significant decline of STH infections in the regions of Littoral, North-West, South and South-West Cameroon in comparison to previous data collected 25 years ago. The results demonstrated the positive impact of annual dewormings of school age children. On the contrary, there was a slight increase of schistosomiasis transmission in several health districts where no mass treatment with praziquantel had been implemented. The study highlighted the need for continuing annual deworming for STH in all districts, and for extending the treatment with praziquantel in those identified moderate and high risk communities for schistosomiasis.

## Competing interests

The authors declare that they have no conflict of interest.

## Authors’ contributions

Conceived and designed the project: LATT, YZ and AT. Analyzed the data: LATT and YZ. Wrote the paper LATT and YZ. Field surveys and data collection: LATT, CDN, PN, ED, CMK and NFG. Provided detailed comments on the draft: AT. Provided leadership for the national control programme: LATT. All authors read and approved the final manuscript.

## Pre-publication history

The pre-publication history for this paper can be accessed here:

http://www.biomedcentral.com/1471-2334/13/602/prepub
